# Normative Data of Extended High Frequency Audiometry in Normal Hearing Subjects with Different Aged Groups

**DOI:** 10.3390/audiolres14060089

**Published:** 2024-12-09

**Authors:** Kittiphorn Luengrungrus, Panida Thanawirattananit, Watchareporn Teeramatwanich

**Affiliations:** Department of Otorhinolaryngology, Faculty of Medicine, Khon Kaen University, Khon Kaen 40002, Thailand; kittiphorn.l@kkumail.com (K.L.); panith@kku.ac.th (P.T.)

**Keywords:** extended high frequency audiometry, normative data, age groups, normal hearing subjects

## Abstract

**Background/Objectives**: Conventional audiometry assesses hearing in the frequency range of 250–8000 Hz, while humans can detect sounds from 20 to 20,000 Hz. Hearing sensitivity above 8000 Hz can be assessed through “Extended high-frequency audiometry (EHFA)”, which is useful for early detection and monitoring of hearing loss and counseling for tinnitus patients. Despite its usefulness, age significantly affects hearing sensitivity. Currently, there are no standard hearing thresholds in the EHF range categorized by age groups in Thailand. In this study, the aim was to determine normative EHFA data across different age groups in a healthy Thai population. **Methods**: This study included 134 participants aged 18–70 years, categorized into five age groups. All participants had normal hearing thresholds and type A tympanograms bilaterally. Following this, EHFA measurement was performed. **Results**: Across all age groups, there was an observed increase in median hearing thresholds as age increased. In comparison, no significant difference in median hearing thresholds was found between individuals aged 18–30 and >30–40 years. However, those over 40 years had significantly higher median thresholds across all frequencies compared to those under 40 years (*p* < 0.05). Based on these findings, we propose two new subgroups of 18–40 years and >40–70 years. Median hearing thresholds for those aged 18–40 was ≤20 dB HL across most frequencies, except at 16,000 Hz (30 dB HL). In the >40–70 age group, thresholds ranged from 20 dB HL at 9000 Hz to 70 dB HL at 14,000 and 16,000 Hz. However, no significant differences were found in hearing thresholds between sexes and ear sides. **Conclusions**: In this study, normative EHFA data were established for healthy Thai adults, appropriately divided into two groups (18–40 and >40–70 years) for clinical use. Further studies with more participants per age group are also needed to validate the results.

## 1. Introduction

Various factors, such as aging, middle ear diseases, ototoxicity, systemic diseases like hypertension and diabetes mellitus, and lifestyle choices, can influence hearing loss [1,2]. Pure tone audiometry is—in conventional audiometry—a hearing test used to measure an individual’s hearing threshold levels ranging from 250 to 8000 Hz, while an individual can detect a signal sound over the frequency range of 20 to 20,000 Hz. Frequencies above 8000 Hz, referred to as “Extended high-frequency (EHF)”, are used for hearing thresholds through “Extended high-frequency audiometry (EHFA)” testing. EHF testing is beneficial for the early detection and monitoring of hearing loss, which often occurs in higher frequencies earlier than in lower frequencies, particularly in patients exposed to ototoxicity [3]. Additionally, EHFA results can guide counseling for patients with tinnitus or hidden hearing loss [1].

EHFA, which utilizes pure-tone stimuli via the air-conduction pathway, is not commonly employed as routine clinical practice for assessing hearing loss. Instead, it uses specific earphones calibrated with reference equivalent threshold sound pressure levels (RETSPLs) within the range of 8000 to 16,000 Hz, in accordance with the ISO 389-8:2004 standard [4]. Despite its usefulness, clinical EHFA lacks age-specific reference standards for diagnostic purposes due to age-related changes in hearing sensitivity. However, studies conducted under ISO 389-5:2006 [5] and ISO 7029:2017 [6] have explored the examination of EHFA.

In terms of EHF hearing loss diagnosis, the ISO 389-5:2006 standard [5] defines a hearing loss at EHF as a hearing level that exceeds 20 dB HL at least once in one or both ears at any frequency. However, this standard is based on participants aged between 18 and 25 years, without distinguishing between genders or further age divisions [5]. As a result, it has limitations in terms of generalizability, meaning that its applicability to broader age groups is restricted, and it lacks gender differentiation. While the ISO 7029:2017 standard [6] recognizes that gender may differently influence EHF hearing loss, it recommends specific equations and coefficients to predict median hearing thresholds between males and females for frequencies of 9000 to 12,500 Hz. However, this criterion is not widely used in practice due to the limited availability of information. Currently, there are significant gaps in knowledge regarding normative data for EHFA across different age groups, particularly in populations over 60 years old. Previous studies on EHFA thresholds have produced inconsistent results, often due to variations in inclusion and exclusion criteria. These criteria frequently fail to account for factors such as differing definitions of normal hearing [7,8,9], the lack of established criteria for systemic diseases [8,9,10] or vascular diseases [7,8,9,10,11], smoking, and alcohol consumption [8,9,10,11]—all of which can impact EHF hearing thresholds. Therefore, establishing normative EHFA thresholds for individuals with normal hearing and no risk factors is essential.

Therefore, acknowledging the significance of EHFA in audiology, in this study, the aim was to determine the normative data hearing thresholds for EHFA in healthy Thai population categorized by age groups. Strict and comprehensive inclusion and exclusion criteria were applied in this current study to ensure accurate and reliable results.

## 2. Materials and Methods

### 2.1. Participants

**Figure 1:**
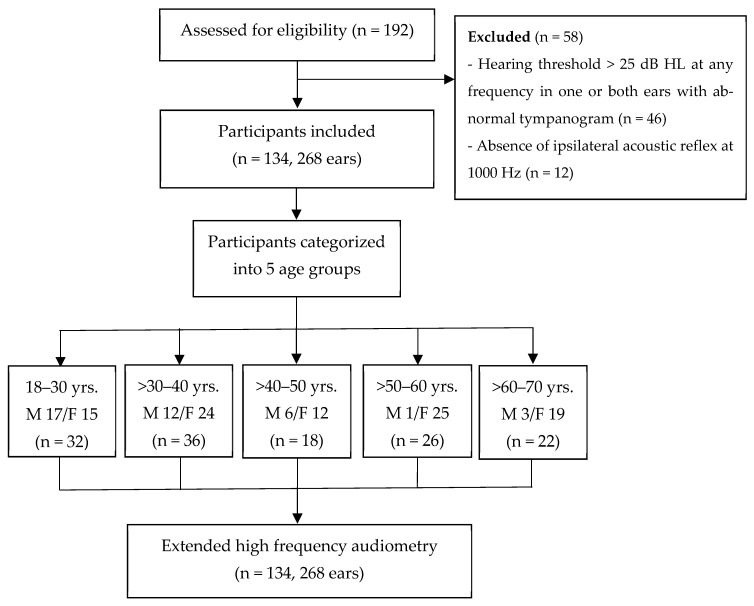
Study population flowchart.

This cross-sectional study involved 134 participants aged between 18 and 70 years, with the sample size calculated and categorized into the following age groups: 18–30 years (32 subjects), >30–40 years (36 subjects), >40–50 years (18 subjects), >50–60 years (26 subjects), and >60–70 years (22 subjects) (Figure 1). This study was conducted at Srinagarind Hospital, Khon Kaen University. Data were collected during September 2023 to February 2024. The inclusion criteria were as follows: (1) Thai individuals aged 18–70; (2) absence of hearing and communication difficulties; (3) no subjective tinnitus; and (4) no history of middle ear problems or surgeries. The exclusion criteria were the following: (1) a history of chemotherapy or aminoglycoside use; (2) occupational noise exposure exceeding 1 year; (3) a family history of hearing loss; (4) presence of systemic diseases; (5) a history of stroke, or chronic kidney disease, or thalassemia with iron chelation; (6) diabetes duration exceeding 5 years [12]; (7) hypertension duration exceeding 3 years or uncontrolled blood pressure levels [13]; (8) smoking exceeding 15 packs/year [14] or continuous smoking exceeding 5 years [15]; (9) alcohol consumption exceeding 400 mL/month; and (10) any abnormal use of drugs. Prior to participation, written informed consent was obtained from all participants after providing information about the hearing examinations and expected benefits.

### 2.2. Ethics Committee

This study received approval from the Khon Kaen University Ethics Committee for Human Research, Faculty of Medicine, on 30 August 2023 (HE661304) and was registered at the Thai Clinical Trials Registry (TCTR20230923002).

### 2.3. Otoscopy Examination and Hearing Evaluation

All subjects underwent an otoscopy examination, and the results were normal. Their hearing thresholds were assessed according to the following procedures: (1) Clinical audiometry using the Interacoustic AC40 audiometer, which measured hearing thresholds from 250 to 8000 Hz via the air-conduction pathway and from 250 to 4000 Hz via the bone-conduction pathway. Subjects exhibiting a hearing level with thresholds exceeding 25 dB HL at any frequency in either ear [16] were excluded, except for those aged >60–70 years, where thresholds at 6000 and 8000 Hz could exceed 25 dB HL as symmetrical hearing between ears; (2) Tympanometry, coupled with ipsilateral acoustic reflex at 1000 Hz, was performed using the GSI 39 Grason-Stadler equipment. Subjects presenting with abnormal tympanometry results or the absence of the ipsilateral acoustic reflex at 1000 Hz were excluded; and (3) EHFA was conducted by an audiologist using the Interacoustic AC40 audiometer to assess hearing thresholds at frequencies of 9000, 10,000, 11,200, 12,500, 14,000, and 16,000 Hz via the air-conduction pathway with Sennheiser HDA300 circum-aural headphones. The maximum sound intensity levels of the equipment are 100–105 dB HL at frequencies of 9000–12,500 Hz, 90 dB HL at 14,000 Hz, and 75 dB HL at 16,000 Hz.

### 2.4. Statistical Analysis

All statistical analyses were performed using IBM SPSS Statistics version 28, with a significance level of 0.05. Descriptive statistics, including frequency, mean, standard deviation, median, and interquartile range (IQR), were employed. To compare hearing thresholds across age groups, one-way ANOVA was employed for parametric data, while the Kruskal–Wallis test was employed for non-parametric data, followed by Bonferroni-adjusted pairwise comparisons. Furthermore, the comparison of hearing thresholds of individuals based on sex and ear were analyzed using t-test for parametric data and Mann–Whitney U test for non-parametric data.

## 3. Results

A total of 192 participants were initially enrolled in this study. However, 58 participants were excluded due to hearing loss at any frequency in one or both ears and abnormal tympanometry results. Consequently, 134 subjects (268 ears) were included in this study, categorized into five age groups. The female-to-male ratio was 2.4:1.

The hearing thresholds across age groups were analyzed. The mean and median values at EHFA for each age group were mostly the same or with differences not exceeding 10 dB. The lowest hearing thresholds mean of 3.44 dB HL (SD = 8.40 dB HL) and median of 5.00 dB HL (IQR = −5.00–5.00 dB HL) were found at the frequency of 9000 Hz in the 18–30 years age group, while the highest hearing thresholds mean of 76.70 dB HL (SD = 7.54 dB HL) and median of 75.00 dB HL (IQR = 70.00–85.00 dB HL) were observed in the oldest aged group (>60–70 years) at the frequency of 14,000 Hz.

**Figure 2:**
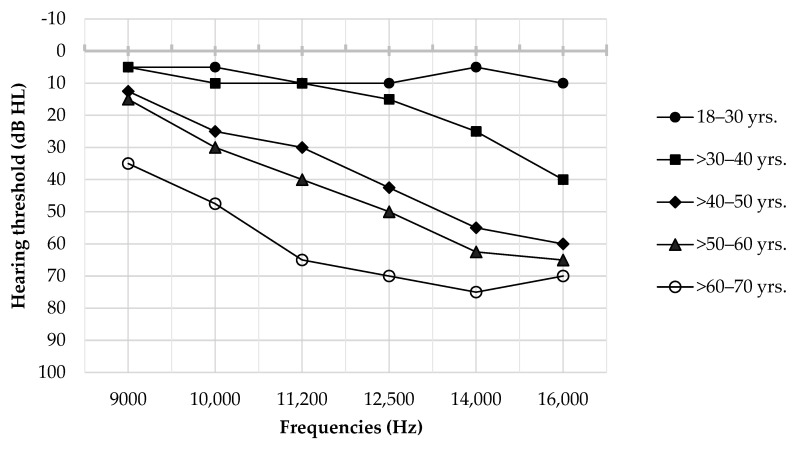
Patterns of median hearing thresholds of EHFA at 9000–16,000 Hz across five age groups.

The patterns of the median hearing thresholds across five age groups are relatively similar at all frequencies. The normative values of median hearing thresholds of the 18–30 and >30–40 age groups showed no significant differences. However, a significant difference in median hearing thresholds emerged starting with the >40–50 age group (Table 1, Figure 2). Furthermore, our results show that the response rate of EHFA remained at 100% across all frequencies in individuals aged 18–70 years, except at the frequency of 16,000 Hz, where it decreased to 79.55%, corresponding to 35 ears out of a total of 44 ears in individuals aged >60–70 years.

**Figure 3:**
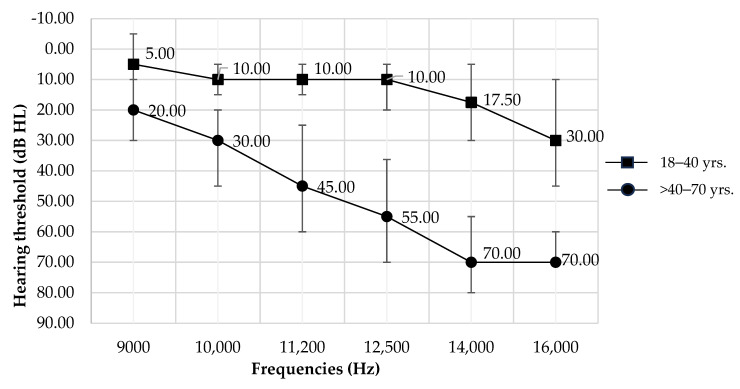
Patterns of median hearing thresholds of EHFA at 9000–16,000 Hz and error bars are included to represent the interquartile range (IQR) by younger (18–40 years) and older (>40–70 years) age groups.

When comparing the median hearing thresholds across five age groups using the Kruskal–Wallis test, we found that individuals younger than 40 years had significantly lower median hearing thresholds at all frequencies (*p* < 0.05) compared to those older than 40 years (Table 1). Therefore, we employed sensitivity analysis by re-categorizing age groups into younger (18–40 years) and older (>40–70 years) age groups for Thai people (Table 2, Figure 3).

In addition, in this study, it is indicated that the hearing thresholds between right and left ears (Supplementary Table S1) and between males and females (Supplementary Table S2) show no significant difference.

## 4. Discussion

This is the first study to establish normative hearing thresholds for extended high frequency audiometry (EHFA) in Thailand, ranging from 9000 to 16,000 Hz. This study included participants aged between 18 and 70 years with normal hearing, selected based on rigorous inclusion and exclusion criteria. Hearing thresholds were specified by sex and by ear side. The findings highlight that the hearing normative thresholds in Thai individuals increase with age. Considering sex and ear side, there are no significant differences in hearing thresholds between males and females and between left and right ears.

In this study, it is indicated that the normative hearing thresholds for EHFA across five age groups were closely aligned with previous findings [9,17]. According to ISO 389–5: 2006 [5], the median hearing threshold for individuals aged between 18 and 25 years is ≤20 dB HL at any frequency. We also confirm that the median hearing thresholds for the youngest Thai age group (18–30 years) are indeed ≤20 dB HL at all high frequency ranges. Specifically, the lowest median hearing threshold is 5 dB HL, while the highest median is 10 dB HL. For those older than 30 years old, the patterns of median hearing thresholds are generally similar for all age groups; the lowest median hearing threshold is observed at the lowest frequency, and the highest median threshold is found at the highest frequency. However, an exception is noted in the >60–70 age group, where the highest threshold is found at the frequency of 14,000 Hz. This could be due to the maximum intensity of the equipment at 14,000 Hz being 90 dB HL, while at 16,000 Hz, the maximum intensity is limited to 75 dB HL. This difference in maximum output levels may affect the measurement of hearing thresholds at 16,000 Hz, leading to a lower threshold value compared to 14,000 Hz.

The current findings also conclude that hearing thresholds in the EHF range in Thailand increase with age, with significant age-related changes becoming evident after the age of 40. This implies that in the Thai population, the effects of aging on hearing begin at frequencies above 9000 Hz starting from this age. Further, sensitivity analysis results suggest that the hearing thresholds for individuals aged 40 years and younger remain ≤20 dB up to 14,000 Hz and 30 dB at 16,000 Hz. For individuals older than 40 years, the hearing thresholds range from 20 dB HL at 9000 Hz to 70 dB HL at 16,000 Hz. This supports earlier studies that have linked age-related changes in the auditory peripheral nervous system [7,8,9], typically starting around the age of 40 and becoming more noticeable at higher frequencies [18].

A Chinese study also indicated that the hearing thresholds at EHF ranges were age-dependent with significant differences beginning at 9000 Hz in the 31–40 years age group [7]. The earlier detection of possible aging effects on changing hearing levels may be attributed to including individuals with clinically hearing thresholds ≤ 20 dB at up to 2000 Hz, while our study included those with hearing thresholds ≤ 25 dB at up to 8000 Hz. Additionally, we conducted both tympanometry testing and tympanometry with acoustic reflex testing to exclude middle-ear pathology. According to the rigorous inclusion and exclusion criteria, our results also highlight that the ability to respond to EHF range dose not decline significantly with age, which is inconsistent with other studies [7,8,9,11,18].

As our study indicates no significant gender-based differences in median hearing thresholds across the five age groups at all frequencies, it implies that hearing median thresholds are similar between males and females. As a result, gender was not a determining factor in hearing sensitivity amongst the Thai population with normal hearing thresholds, especially in the EHF range up to 16,000 Hz. Therefore, EHFA may not need to be gender-specific. Previous studies also found similar hearing thresholds for males and females, especially in the younger age groups [7,8,19]. However, some studies [9,17] investigated the impact of gender on hearing loss in the EHF range, comparing median hearing thresholds to the ISO 7029:2017 standard [6]. Our findings reveal lower median hearing thresholds that closely align with the ISO 7029 standard [6] for both sexes across all age groups. This provides clear evidence that the median EHFA thresholds derived from this study are reliable and applicable for practical use. Moreover, a prior study in the Czech Republic suggested that for individuals over 30 years of age, differences in hearing thresholds between sexes could be observed at frequencies of 11,200 and 12,500 Hz. Furthermore, as age increased, differences in hearing thresholds between sexes became evident, beginning at lower frequencies around 9000 Hz when compared to individuals under 30 years old. It is worth noting that there were no significant differences in hearing thresholds between sexes at the frequencies of 14,000 and 16,000 Hz [8]. The differences in hearing thresholds between sexes may occur due to several risk factors that may vary between sexes such as exposure to noise, smoking, alcohol consumption [7,11], and underlying health conditions [7] that were controlled in this study through strict exclusion criteria.

Furthermore, the study found no statistically significant differences in EHFA hearing thresholds between the right and left ears. This finding is consistent with previous studies indicating that individuals with normal hearing are able to detect the sound by right and left ear similarly [7,20,21]. However, it is evident that individuals who have ear diseases can develop an asymmetrically hearing between two ear sides [22].

In this study, internal validity was prioritized by applying stringent criteria to include a healthy population. This may limit its generalizability to the broader populations, especially those with underlying conditions such as diabetes, hypertension, and those with high-risk behaviors like smoking and alcohol consumption. To address this limitation, we recommend that future studies compare normative hearing thresholds between individuals with normal hearing with and without hearing loss risk factors. This may enhance the applicability of the findings.

## 5. Conclusions

This is the first study in Thailand that attempts to provide valuable insights into the normative data of EHFA hearing assessments, categorized by age group. The results conclude that standardizing EHFA thresholds requires appropriate age group divisions for healthy Thai populations without hearing risk factors. The most suitable age ranges for establishing median and interquartile normative data of EHFA are 18–40 years and >40–70 years. The EHFA normative data identified by this current study have strong internal validity due to the establishment of clear inclusion and exclusion criteria, enhancing the credibility of the findings. Therefore, the median and interquartile range of EHFA thresholds can be applied clinically, and other future studies can use our results as a benchmark for their results. Furthermore, we also confirm that sex and ear side are not determining factors for establishing hearing thresholds among healthy populations. However, it is essential to recognize the limitation of this study regarding generalizability, as it was conducted within a healthy population and may not accurately reflect the characteristics of Thai individuals at risk of hearing loss.

## Figures and Tables

**Table 1:** Median and interquartile hearing thresholds of EHFA at 9000–16,000 Hz across age groups.

Frequency (Hz)	Median of EHF Threshold (dB HL) (Q1, Q3)				
	18–30 yrs. (64 Ears)	>30–40 yrs. (72 Ears)	>40–50 yrs. (36 Ears)	>50–60 yrs. (52 Ears)	>60–70 yrs. (44 Ears)
9000	5.00 (−5.00, 5.00)	5.00 (−3.75, 10.00)	12.50 * ^∆^ (10.00, 20.00)	15.00 * ^∆^ (10.00, 25.00)	35.00 * ^∆^ (20.00, 43.75)
10,000	5.00 (1.25, 13.75)	10.00 (5.00, 15.00)	25.00 * ^∆^ (15.00, 33.75)	30.00 * ^∆^ (16.25, 35.00)	47.50 * ^∆^ (35.00, 60.00)
11,200	10.00 (5.00, 15.00)	10.00 (5.00, 20.00)	30.00 * ^∆^ (21.25, 45.00)	40.00 * ^∆^ (25.00, 53.75)	65.00 * ^∆^ (55.00, 70.00)
12,500	10.00 (0.00, 18.75)	15.00 (5.00, 25.00)	42.50 * ^∆^ (25.00, 58.75)	50.00 * ^∆^ (35.00, 65.00)	70.00 * ^∆^ (65.00, 80.00)
14,000	5.00 (0.00, 20.00)	25.00 (15.00, 40.00)	55.00 * ^∆^ (35.00, 70.00)	62.50 * ^∆^ (50.00, 75.00)	75.00 * ^∆^^■^ (70.00, 85.00)
16,000	10.00 (0.00, 35.00)	40.00 (30.00, 48.75)	60.00 * ^∆^ (50.00, 70.00)	65.00 * ^∆^ (60.00, 70.00)	70.00 * ^∆^ (70.00, 75.00)

Q1: 25th quartile; Q3: 75th quartile. * Statistically significant difference (*p* < 0.05) from aged 18–30 years. ^∆^ Statistically significant difference (*p* < 0.05) from aged >30–40 year. ■ Statistically significant difference (*p* < 0.05) from aged >40–50 years.

**Table 2:** Median and quartile hearing thresholds of EHFA at 9000–16,000 Hz by younger (18–40 years) and older (>40–70 years) age groups.

Frequency	Median of EHF Threshold (Q1, Q3) in dB HL	
(Hz)	18–40 yrs. (136 Ears)	>40–70 yrs. (132 Ears)
9000	5.00 (−5.00, 10.00)	20.00 (10.00, 30.00)
10,000	10.00 (5.00, 15.00)	30.00 (20.00, 45.00)
11,200	10.00 (5.00, 15.00)	45.00 (25.00, 60.00)
12,500	10.00 (5.00, 20.00)	55.00 (36.25, 70.00)
14,000	17.50 (5.00, 30.00)	70.00 (55.00, 80.00)
16,000	30.00 (10.00, 45.00)	70.00 (60.00, 70.00)

Q1: 25th quartile; Q3: 75th quartile.

## Data Availability

The data supporting the findings of this study are available from the corresponding author, Watchareporn Teeramatwanich, upon reasonable request.

## Supplementary Materials

The following supporting information can be downloaded at: https://www.mdpi.com/article/10.3390/audiolres14060089/s1, Table S1: mean and standard deviation of EHFA thresholds by frequency and ear across age groups; Table S2: mean and standard deviation of EHFA thresholds by frequency and sex across age groups.
