# Supplementary material for: Normative Data of Extended High Frequency Audiometry in Normal Hearing Subjects with Different Aged Groups
Source: Audiol Res. 2024 Dec 9;14(6):1084–92. doi: 10.3390/audiolres14060089 (PMC11674001; doi:10.3390/audiolres14060089)
Supplement: Supplementary file 1 [file audiolres-14-00089-s001.zip › audiolres-3302015-supplementary.pdf]

Table S1. Mean and standard deviation of EHFA thresholds by frequency and ear across age groups

Frequency (Hz)	Mean and standard deviation of EHFA thresholds														
	18–30 yrs.			>30–40 yrs.			>40–50 yrs.			>50–60 yrs.			>60–70 yrs.		
	Right	Left	p-value	Right	Left	p-value	Right	Left	p-value	Right	Left	p-value	Right	Left	p-value
9000	3.28 (7.99)	3.59 (8.91)	.896	3.61 (9.15)	5.56 (8.09)	.199	12.50 (7.52)	16.11 (8.67)	.214	16.73 (7.99)	19.42 (10.61)	.418	32.05 (16.45)	31.14 (15.50)	.943
10,000	7.19 (7.92)	7.66 (8.89)	.874	10.14 (8.90)	10.97 (6.64)	.570	22.78 (10.46)	26.11 (12.07)	.424	26.54 (15.48)	27.69 (13.58)	.919	47.50 (15.33)	44.55 (20.00)	.705
11,200	8.75 (7.83)	8.91 (9.90)	.864	11.53 (10.06)	13.19 (8.21)	.253	33.61 (14.43)	35.00 (17.15)	.938	38.08 (18.71)	39.23 (16.04)	.755	59.55 (15.73)	61.14 (18.89)	.478
12,500	8.13 (11.83)	10.94 (13.16)	.435	15.28 (11.14)	15.56 (10.87)	.896	42.50 (20.45)	42.22 (20.95)	.913	49.04 (17.61)	50.58 (19.56)	.818	69.77 (14.27)	69.32 (16.21)	.840
14,000	10.78 (14.71)	11.41 (15.77)	.919	26.67 (15.63)	28.19 (15.50)	.743	53.06 (19.79)	50.83 (21.57)	.815	63.27 (-13.56)	61.92 (14.15)	.761	77.27 (7.67)	76.14 (7.55)	.542
16,000	16.88 (19.83)	15.63 (21.13)	.700	37.08 (14.85)	38.19 (13.84)	.691	58.61 (13.15)	58.61 (14.02)	.963	64.80 (7.97)	64.81 (7.14)	.938	70.59 * (3.48)	71.39 * (3.76)	.443

Note: * For >60–70 age group at frequency 16,000 Hz, the mean hearing thresholds were calculated based on those with hearing test response only (right ear = 17 ears, and left ear = 18 ears)

Table S2. Mean and standard deviation of EHFA thresholds by frequency and sex across age groups

Frequency (Hz)	Mean and standard deviation of EHFA thresholds								
	18–30 yrs.			>30–40 yrs.			>40–50 yrs.		
	Male	Female	p-value	Male	Female	p-value	Male	Female	p-value
9000	5.74 (7.79)	0.83 (6.86)	.069	3.96 (7.03)	4.90 (8.02)	.830	14.58 (8.43)	14.17 (7.49)	.892
10,000	10.15 (7.93)	4.33 (6.97)	.053	11.25 (6.70)	10.21 (6.75)	.830	25.42 (11.34)	23.96 (10.42)	.820
11,200	9.85 (9.21)	7.67 (7.16)	.526	11.67 (10.19)	12.71 (7.03)	.497	31.25 (17.23)	35.83 (14.51)	.494
12,500	11.47 (13.35)	7.33 (10.24)	.433	16.67 (11.89)	14.79 (9.26)	.608	34.58 (25.52)	46.25 (17.11)	.250
14,000	15.29 (16.37)	6.33 (9.90)	.089	25.42 (15.66)	28.44 (14.67)	.679	41.67 (23.59)	57.08 (17.54)	.151
16,000	20.44 (21.91)	11.5 (16.06)	.331	39.79 (12.36)	36.56 (14.31)	.476	52.08 (18.13)	61.88 (9.54)	.291

This analysis excluded data from individuals over the age of 50 due to a lower number of male participants compared to female participants. Consequently, EHFA data collected was insufficient for meaningful analysis.
